# Supplementary material for: Light-responsive transcription factor CmOzf integrates conidiation, fruiting body development, and secondary metabolism in Cordyceps militaris
Source: Microbiol Spectr. 2025 Oct 20;13(12):e01057-25. doi: 10.1128/spectrum.01057-25 (PMC12671173; doi:10.1128/spectrum.01057-25)
Supplement: Supplemental material — Fig. S1 to S7; Table S1. [file spectrum.01057-25-s0001.docx]

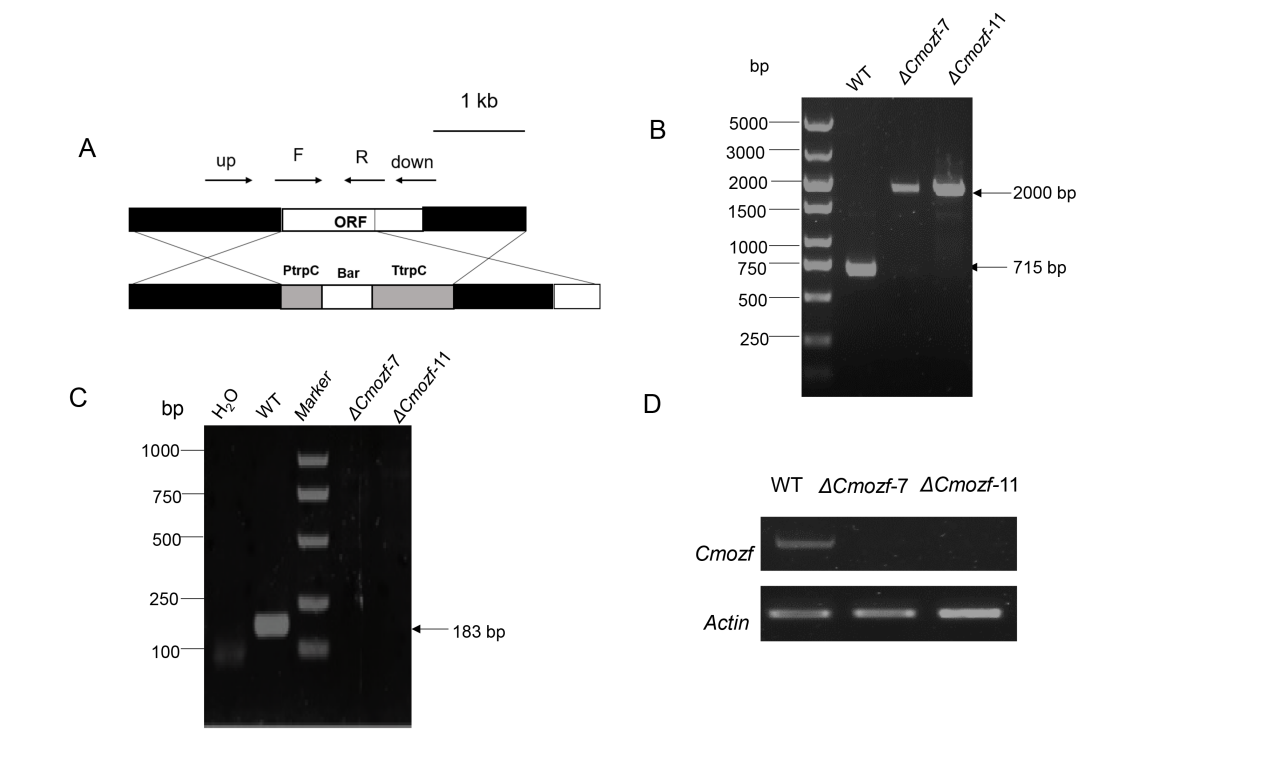


**FIG. S1**  Identification of *Cmozf* gene knockout transformants. (A) Schematic of *Cmozf* gene knockout. PCR identification was performed using primers *Cmozf*-up/down from the flanking regions (B) and *Cmozf*-F/R located in the deletion region (C). (D) RT-PCR was conducted to determine gene expression in the knockout mutants, and the *actin* gene was used as a reference. *ΔCmozf*-7 and *ΔCmozf*-11 represented two *Cmozf* deletion transformants, respectively.


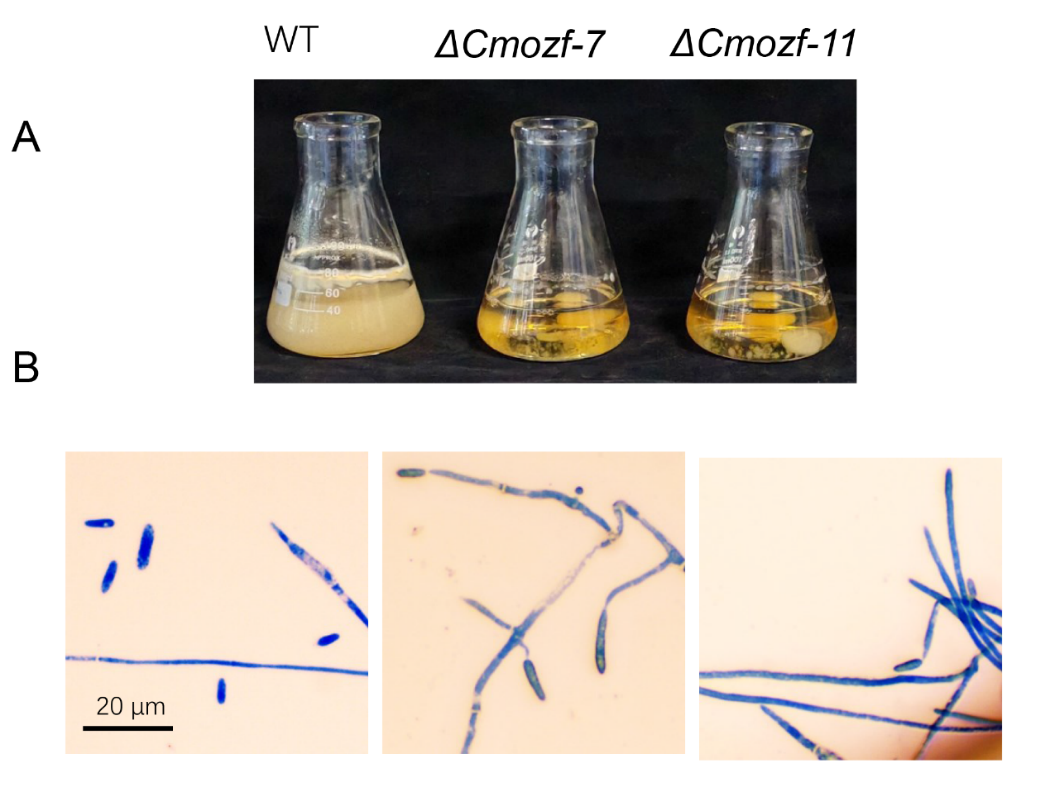


**FIG. S2** Colony morphology of the *Cmozf* knockout mutant cultured in 1/4 SDB medium for 4 days. (A) Photograph taken after 4 days of culture in 50 mL of 1/4 SDB medium; (B) Observation of blastospore formation under a light microscope.


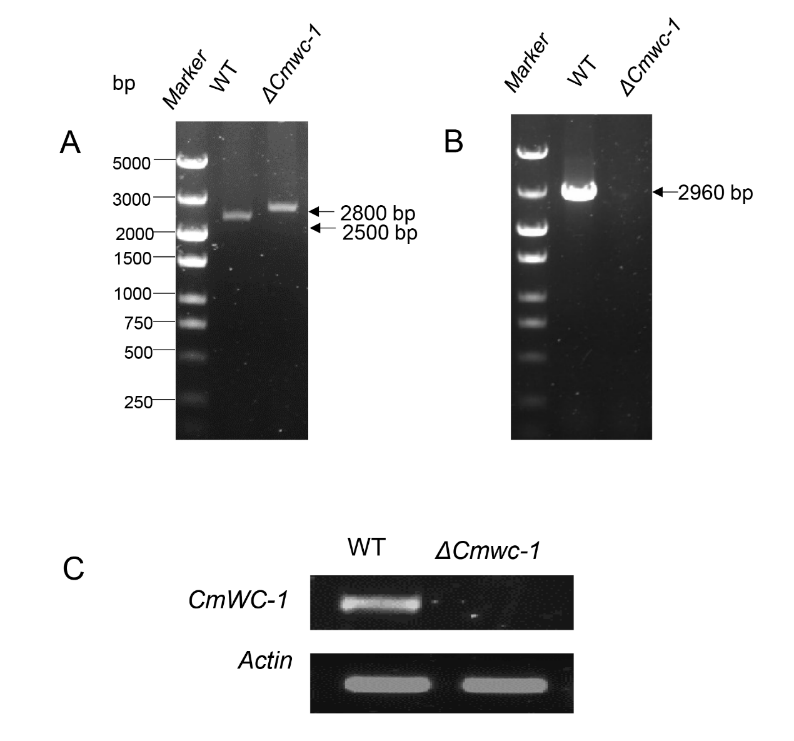


**FIG. S3** Disruption of *Cmwc-*1. (A) PCR analysis was conducted to confirm the deletion of the *Cmwc-1* gene, using primers located in the flanking regions of the gene. (B) PCR was performed using primer pairs specific to the open reading frame (ORF) region of *Cmozf*, and no fragment was observed in the mutant strain. (C) RT-PCR analysis of *ΔCmwc-1* mutant, *Cmwc-1* expression was not detected in the *ΔCmwc-1* mutant.


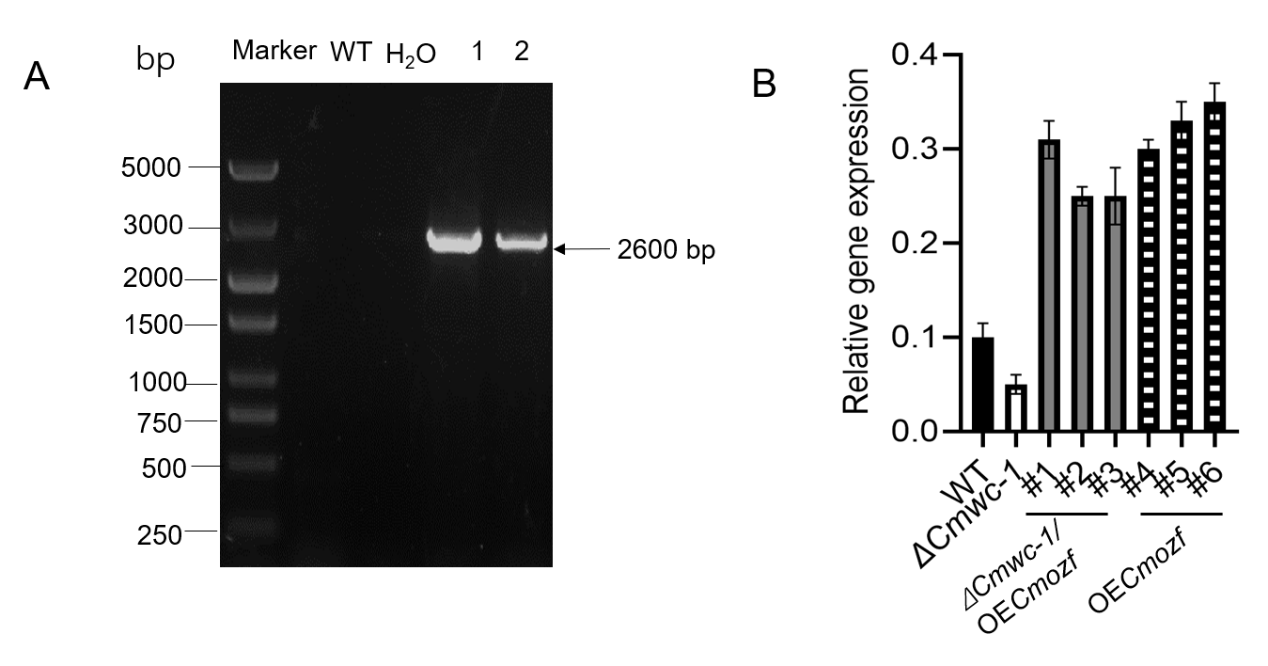


**FIG. S4** Screening of overexpression transformants. (A) PCR validation of overexpressing transformants using primers *pb3*-F/*pb3*-OE*Cmozf*-R with genomic DNA as template. 1: *ΔCmwc-1/*OE*Cmozf* ; 2: OE*Cmozf.* (B) qRT-PCR analysis using cDNA from overexpressing transformants to determine transcript levels, with the strain exhibiting the highest gene expression selected for subsequent experiments. #1- #3: *ΔCmwc-1/*OE*Cmozf* ; #4 - #6: OE*Cmozf*.


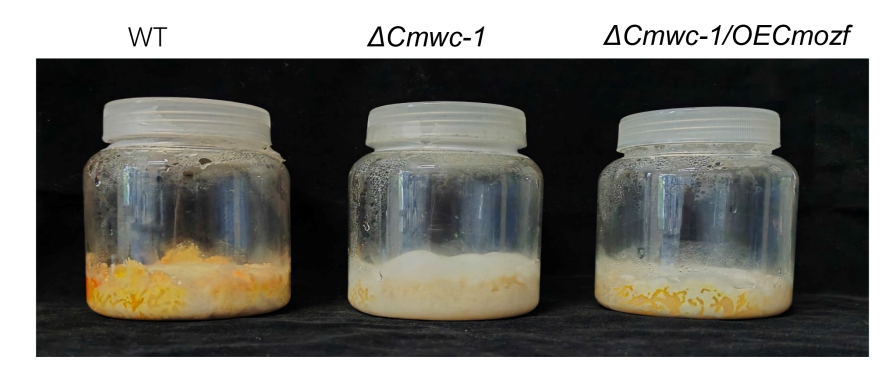


**Fig. S5.** The undifferentiated primordium of the *ΔCmwc-1*/OE*Cmozf* strain.
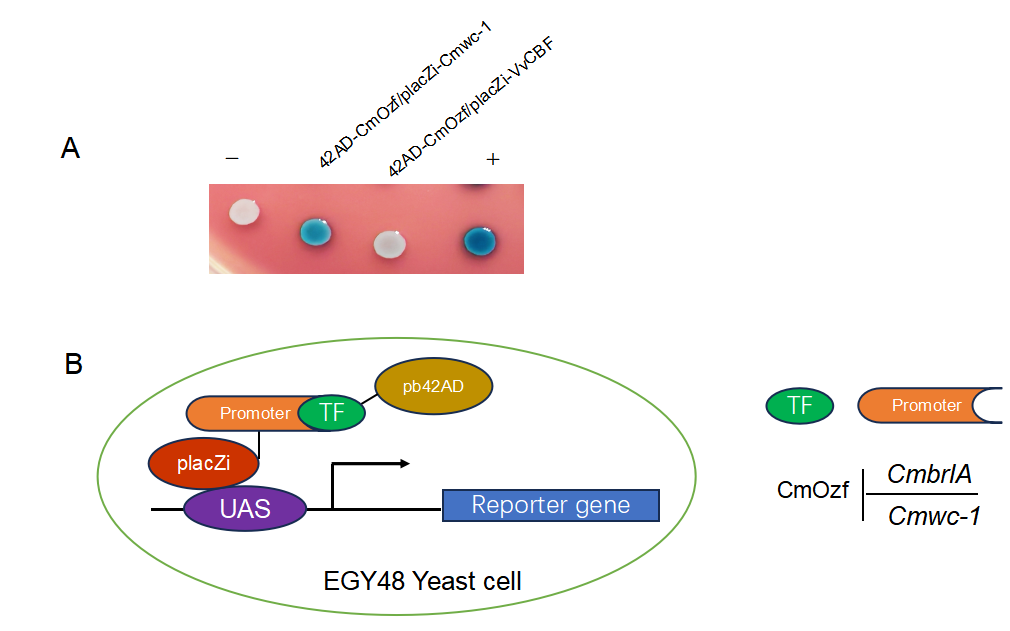


**FIG. S6** Binding analysis of transcription factors to target promoters. (A)Yeast one-hybrid assay showing the interaction between transcription factor CmOzf and the *Cmwc-1* promoter. The empty pLacZi vector and pLacZi-*Vvcbf* promoter were used as negative controls. (B) Schematic diagram of the transcription factor-promoter interaction.


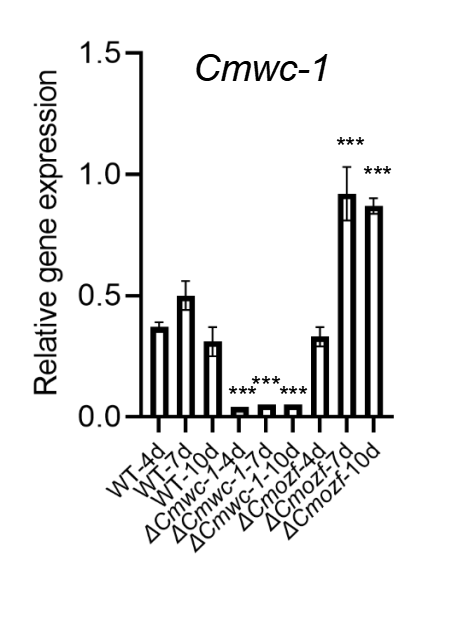

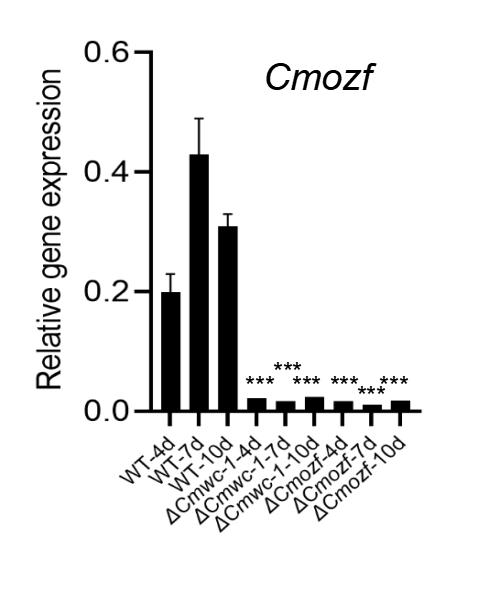
A B

**FIG. S7** mRNA levels of *Cmozf* (A) and *Cmwc-1* (B) in *ΔCmwc-1* and*ΔCmozf* strains. Samples were collected after 3 days of dark incubation followed by 1, 4, and 7 days of continuous light for 1, 4, and 7 days on PDA medium. Asterisks indicate statistically significant differences in gene expression between mutant and WT strains at same developmental stages: ***: *P* < 0.001.

**TABLE S1** Primers in this study

|  | For RT-PCR |
| --- | --- |
| *qCmozf*-F/R | TCGCAATGACTACCAGGAGC//GTAATCGTTTTGGCGGAGGC |
| qCmwc-1-F/R | TCCTGATGCCATTGCTGGTT//GATGCCAGTCTGACGCGATA |
| qCmvvD-F/R | TACGCGCCTAGTGGGATAGA//GGAAAAAGCCTCGTTTGCGT |
| qCmbrlA-F/R | TTCATGATGACGCCCACACA//GGAGGAGCACCCTGTAGGTA |
| qCmabaA-F/R | ATTGAGCAGATGGGACGCAA//CTGTGAGGATGGGGTTGGAC |
| qCmwetA-F/R | CAGCCCGATCTCTTTTCCCA//GCAGGGTTGGAGGACAAGAA |
| q00301-F/R | CGAAAAGTCCCTGACCGACA//ATGCGCTTACCCTCAAGGTC |
| q04199-F/R | GAGCAATGCGCGCTGAATAA//CTCGTATTCCCACTCGGACG |
| q09155-F/R | CAAGTGCAAGTTTGAGGCCC//CCGATGATGAGGATGGAGCC |
| q00637-F/R | GTCCCCGTGACTTGGATGTT//ATCCCACCAGAGTAGACCCC |
| q09317-F/R | GCGCTGTGAAAGAACAGCAA//TGGTCAATGCAGGGCAGAAT |
| Cmactin-F/R | GCCGAGGAAACAACAGAA//GCAGTCGTGGCAAGGAT |
|  | For mutant strains |
| *Cmozf*-left-F | CAGCTATGACCATGATTACGAATTCCTGAGACTCCCTCTGGTATC |
| *Cmozf*-left-R | TCTTCTGTCGACACTAGTGAATTCAGAAAGGCGGAGGGAATGAG |
| *Cmozf*-right-F | TTAGAGGTAATCCTTCTTTCTAGACATGATTTTCCAGCGCGACT |
| *Cmozf*-right-R | GCATGCCTGCAGGTCGACTCTAGATGAAAAAGAGGCGGCGCAGT |
| *Cmozf*-up/down | GCCTCTACAAAACATCTCGA//GTAATCGTTTTGGCGGAGGC |
| *Cmwc1*-left-F | CAGCTATGACCATGATTACGAATTCGGTGGTACGTTGCCAGTTCT |
| *Cmwc1*-left-R | TCTTCTGTCGACACTAGTGAATTCACCCAGAAACGCTACGACCT |
| *Cmwc1*-right-F | TTAGAGGTAATCCTTCTTTCTAGACTCTATGAACGACTGCCCGA |
| *Cmwc1*-right-R | GCATGCCTGCAGGTCGACTCTAGATCAGACTCAAGTCGAGCTAG |
| *Cmwc1*-up/down | TTCTACATAGGTACCTAGGT//TGGTCGCATCTTTCAACTCT |
| Cmwc1-F/R | ATGGAAGGCTACTATCCTCC//TCAAGTCGAGCTAGTCTCTC |
| For over expression genes | |
| pb3-F | CTATGACCATGATTACGAATTCGTTGGGTATGCTCCGG |
| *pb3*-OECmozf-R | TTGAGCAGTGAGAGCCATTGTTATTGATTAAAAGGGT |
| OECmOzf-F | ACCCTTTTAATCAATAACAATGGCTCTCACTGCTCAA |
| OECmOzf-R | TCTTCTGTCGACACTAGTGAATTCTTAGTACAGGCGGTCGGTGA |
| For yeast one-hybrid assay | |
| *Cmozf*-42AD-F | TGCCTCTCCCGAATTCATGGCTCTCACTGCTCAACC |
| *Cmozf*-42AD-R | AGTCCAAAGCTTCTCGAGTTAGTACAGGCGGTCGGTGA |
| *Cmwc1*-42AD-F | TGCCTCTCCCGAATTCATGGAAGGCTACTATCCTCC |
| *Cmwc*1-42AD-R | AGTCCAAAGCTTCTCGAGTCAAGTCGAGCTAGTCTCTC |
| pLacZi*-CmbrlA*-F | ATTGAAAAGCTTGAATTCTTTCCCCCTCCCTTGAACCT |
| pLacZi-CmbrlA-R | GAGCACATGCCTCGAGTGTGGTGGCCATTGAGCAAG |
| Placz-*Cmwc1*-F | ATTGAAAAGCTTGAATTCTTCTACATAGGTACCTAGGT |
| Placzi-*Cmwc1*-R | GAGCACATGCCTCGAGCGGCGGCATCTAGACAGTAT |
| Placzi-*Cmwc1*-P1-R | GAGCACATGCCTCGAGTACGCATCGCGCACCACTAG |
| Placzi-*Cmwc1*-P2-F | ATTGAAAAGCTTGAATTCGCGATGCGTAATCTTTATTG |
| Placzi-*Cmwc1*-P2-R | GAGCACATGCCTCGAGGCGGCAGATTGGTATTGAAG |
| Placzi-*Cmwc1*-P3-F | ATTGAAAAGCTTGAATTCCAATCTGCCGCAGCTCAATT |
